# Proximate, Physicochemical, Techno-Functional and Antioxidant Properties of Three Edible Insect (*Gonimbrasia belina*, *Hermetia illucens* and *Macrotermes subhylanus*) Flours

**DOI:** 10.3390/foods11070976

**Published:** 2022-03-28

**Authors:** Nthabeleng Vanqa, Vusi Vincent Mshayisa, Moses Basitere

**Affiliations:** 1 Department of Food Science and Technology, Cape Peninsula University of Technology, Bellville 7535, South Africa; vanqanthabeleng@gmail.com; 2 Academic Support Program for Engineering (ASPECT) Cape Town, Centre of Higher Education Development University of Cape Town, Rondebosch, Cape Town 7701, South Africa; moses.basitere@uct.ac.za

**Keywords:** edible insect flours, *G. belina*, *H. illucens*, *M. subhylanus*, nutritional properties, techno-functional properties, antioxidant activity, metal chelation, Mashonzha, Madzhulu, black soldier fly

## Abstract

In this study, edible insect flours from *Gonimbrasia belina* (Mashonzha), *Hermetia illucens* (black soldier fly larvae) and *Macrotermes subhylanus* (Madzhulu) were prepared and assessed in terms of proximal, physicochemical, techno-functional and antioxidant properties. The crude protein of the edible insect flours varied between 34.90–52.74%. The crude fat of the insect flours differed significantly (*p* < 0.05), with *H. illucens* (27.93%) having the highest crude fat. *G. belina* was lighter (L*) and yellower (+b*) compared to *H. illucens* and *M. subhylanus*, and there was no significant difference (*p* > 0.05) in the redness (+a*) of the edible insect flours. There were no significant differences (*p* > 0.05) in foam capacity and foam stability of all three edible insect flours. Moreover, the antioxidant activity against the DPPH radical was low for *H. illucens* (3.63%), with *M. subhylanus* (55.37%) exhibiting the highest DPPH radical. Principal component analysis (PCA) was applied to the techno-functional properties and antioxidant indices of the edible insect flours. PC1 accounted for 51.39% of the total variability, while component 2 accounted for 24.71%. In terms of PC1, the FS, OBC and FC were responsible for the major differences in the edible insect flours. The findings revealed that edible insect flours are a good source of antioxidants and can be used as an alternative protein source and a potential novel food additive due to their techno-functional qualities.

## 1. Introduction

As vast as the challenge is to feed 9 billion people by 2050, increasing food availability is insufficient due to the increasingly limited resources, such as agriculturally cultivable land [1]. This, without a doubt, calls for innovative, alternative ways of ensuring that adequate, quality, safe and nutritious foods are available and accessible to all people at all times [2]. As early as 1975, Meyer-Rochow [3] argued and proposed that edible insects could play a role in alleviating food security and combating protein deficiency in some underdeveloped countries. Over the last two decades, there has been a renewed interest on edible insects for human consumption globally [4,5,6]. The FAO report titled “*Edible insects: Future prospects for food and feed*” [6] and other scientific literature seems to have re-invigorated the earlier call made by Meyer-Rochow in 1975. This is because, compared to conventional protein sources, edible insects have an excellent feed conversion ratio; a source of protein, fat and minerals, and this characteristic is particularly valuable given that future protein consumption is expected to increase with a declining food supply [7,8,9,10].

Entomophagy, the practice of consuming insects, has been practised worldwide for centuries, yet it has only recently gained momentum in Western cultures [11]. Insects are consumed prominently in Latin America, Asia, and Africa [10]. People throughout the world have been consuming insects as a regular part of their diets for millennia [12]. Considering the growing population worldwide and the increasing demand for additional sources of proteins, edible insects are seen as an economical alternative and as a sustainable source of nutrients and bioactive compounds [13]. *Hermetia illucens* (black soldier fly), *Gonimbrasia belina* (Mashonzha) and *Macrotermes subhylanus* (Madzhulu) are among edible insect species that have gained attention as alternative sources of protein; the latter two are indigenous to parts of South Africa and play a vital role in food security, rural livelihoods, and poverty eradication [4]. Black soldier fly larvae are commercially produced in South Africa by one of the largest industrial insect processing companies, AgriProtein. The European Food Safety Authority (EFSA) is currently considering black soldier fly as a novel ingredient to be used in food.

*Gonimbrasia belina* (*G. belina*) is an emperor moth species indigenous to Southern Africa’s warmer areas. It is a giant edible caterpillar, known as the Mashonzha (in Venda), madora (in Shona) or mopane worm or amacimbi (Ndebele), which mainly feeds on mopane tree leaves but not exclusively. For millions in the region, Mashonzha are a significant source of protein. Emperor moth *G. belina* caterpillars are a significant natural resource for rural individuals residing in Botswana, Namibia, northern South Africa, and southern Zimbabwe’s mopane forests [14].

*Macrotermes subhylanus* (*M. subhylanus*), known as Madzhulu in Venda and isusu in Nigeria are termites and are gregarious insects most common during the rainy season [4,15]. They are the second most eaten insects in South Africa and are harvested during the rainy season. At the same time, Mashonzha and Madzhulu are sold at informal markets predominantly in the Limpopo and KwaZulu Natal provinces, and in other parts of South Africa are considered a delicacy.

In addition to insects, algae and in vitro meat have also been considered as potential alternatives to conventional sources [16]. The inclusion of insects among these alternatives is highly recommended since they are widely incorporated in food cultures worldwide and have excellent nutritional qualities.

Nevertheless, it is essential to highlight that food neophobia is still directed to the consumption of edible insects, especially in western and urban societies. However, Schösler et al. [17] reported that edible insects, if incorporated in foods in a less obvious form, such as food ingredients (flours, powders, or pastes) in products that are indistinguishable from familiar food items, consumers would accept them. This indicates that insects could be used as food ingredients in the food supply chain, particularly in areas where traditional approaches are unlikely to be adopted owing to a lack of sensory appeal, and insect flour is one way to incorporate insects into food production systems

Therefore, it is crucial to note that the first step to large-scale industrial success is the exploration of the nutritional, techno-functional and antioxidant properties of proposed edible insect ingredients. Currently, available literature on the application of insect flour mainly focuses on *T. molitor (mealworm*) [13,18]. There has been little attention paid to the nutritional, techno-functional and antioxidant properties of Mashonzha, black soldier fly larvae and Madzhulu edible insect flours from South Africa.

Therefore, the aim of this study was to establish the proximate composition, physicochemical, techno-functional properties, and antioxidant activity of edible insect flours obtained from Mashonzha, black soldier fly larvae and Madzhulu with the view to find alternative protein sources for human consumption.

## 2. Materials and Methods

### 2.1. Source of Materials

The edible insects were sourced from different provinces of South Africa: Mashonzha (*G. belina*) and Madzhulu (*M. subhylanus*) were sourced in the Vhembe district, Limpopo, and the black soldier fly larvae (*H. illucens*) was sourced from Cape Town, Western Cape, South Africa. The chemical reagents, 2,2 diphenyl-1-picrylhydrazyl (DPPH), 2,2′ azobis (2-methyl, 2,2-Azinobis (3-ethylbenzothiazoline-6-sulphonic acid) diammonium salt (ABTS), Ferric (III) chloride, ethylenediaminetetraacetic acid (EDTA), tertiary butyl hydroquinone (TBHQ), ferrous (II) chloride and thiobarbituric acid (TBA) were obtained from Merk (Sigma-Aldrich, Kempton Park, South Africa). All the chemicals used in this study were of analytical grade, and chemical reagents were prepared according to standard analytical procedures. Prepared reagents were stored under conditions that prevented deterioration or contamination. The water used in the study was ultrapure water purified with a Milli-Q water purification system (Millipore, Microsep, Bellville, South Africa). The ethics committee of the faculty of applied sciences gave its approval to the study (215062965/05/2021).

### 2.2. Preparation of Insect Flours

Representative samples of sun-dried Mashonzha (hereinafter indicated as *G. belina*) and Madzhulu (hereinafter indicated as *M. subhylanus*) edible insects were purchased from street vendors from the Vhembe district (Limpopo province, South Africa). Black soldier fly larvae (hereinafter indicated as *H. illucens*) reared on clean larvae was purchased from AgriProtein (Cape Town, South Africa), and the flour was prepared following the method described by Zozo et al. [19] and freeze-dried (Wizard 2.0, SP Scientific, Johannesburg, South Africa). The dried edible insects were subjected to a grinding/milling process using a laboratory blender (Bamix, Checkers, Cape Town, South Africa). The flours were stored at room temperature under conditions that prevented deterioration.

### 2.3. Proximate Composition Analysis

Proximate composition, i.e., moisture (925.10), crude protein (920.87), crude fat (932.06), and ash content (923.03) of the insect flours were determined following standard methods recommended by the Association of Official Analytical Chemists (AOAC) [20]. The crude protein determination was performed using Dumas (TruSpec^™^ Leco Carbon/Hydrogen/Nitrogen Series, Leco Africa) which was calibrated with EDTA according to Zozo et al. [19]. The crude protein was subsequently calculated by multiplying nitrogen content by a protein-to-nitrogen conversion factor of 5.60 as recommended by Janssen et al. [21]. Moisture percentage was calculated by drying the sample in a vacuum oven at 100 °C for two hours. The dried sample was placed into a desiccator, allowed to cool, and then re-weighed. The process was repeated until a constant weight was obtained. Crude fat was calculated by drying fats after extraction in a Soxhlet assembly using petroleum ether. The ash percentage was calculated by combusting the samples in a silica crucible placed in a muffle furnace at 550 °C. The percentage of carbohydrates on a dry basis was determined by subtracting all the components (moisture, crude protein, crude lipid, and ash) from 100. The energy was calculated using the formula [22]:

$$\mathrm{Energy}\;\left(\frac{\mathrm{Kcal}}{100g}\right)=4\;\left(\%\;\mathrm{Carbohydrates}+\%\;\mathrm{Protein}\right)+\left(9\times \;\%\mathrm{fat}\right)$$

### 2.4. Determination of Physicochemical Properties

#### 2.4.1. Evaluation of Colour Properties of Edible Insect Flours

The colour of the edible insect flours was measured using spectrophotometry (Model CM-5, Konica Minolta Sensing, Tokyo, Japan) as described by [23], set at standard observer 10° and D65. The instrument was zero calibrated using a black tile (L* = 5.49, a* = −7.08, b* = 4.66) and white calibration was performed using a white tile (L* = 93.41, a* = −1.18, b* = 0.75). Edible insect flour samples were evenly placed in a petri-dish (30 mm diameter), and reflectance was measured for L*a*b* colour scales. The L* coordinate is lightness, 100 represents white and closer to 0 represents black Measurements for each sample were performed in triplicate at three different positions in the samples, with the results recorded in L* (lightness), a* (chromaticity coordinate +a* = red and −a* = green), b* (chromaticity coordinate +b* = yellow and −b* = blue).

#### 2.4.2. Determination of Bulk Density

The procedure was described by Mintah et al. [24] with some modifications. First, 5 g of the sample was transferred into a weighed measuring cylinder (50 mL) ($W_{1})$ and then compressed by tapping until sample volume remained constant. The tube was again weighed ($W_{2}$) the new volume ($V_{1}$) was noted and the density (g/mL) was measured using the following formula:

$$\mathrm{Bulk}\;\mathrm{Density}=\frac{W_{2}-W_{1}}{V1}$$

#### 2.4.3. Determination of Water Activity

The water activity (A_w_) of edible insect flours was measured using the method described by Benamara et al. [25] with minor modifications. Salt humidity standards of 53, 75 and 90% relative humidity were used to calibrate the measurement cell. A sample (5 g) of the insect flours was transferred into a sample dish and placed inside the (AW SPRINT TH500, Novasina analyser, Zurich, Switzerland), and the cell measuring protection filter was immediately closed. The reading was observed after a period of 60 to 80 s.

### 2.5. Determination of Techno-Functional Properties

#### 2.5.1. Determination of Water Binding Capacity and Oil Binding Capacity

The water-binding capacity (WBC) of the edible insect flours was determined according to Mshayisa and van Wyk [26] with slight modifications. Briefly, a 0.5 g sample was mixed with 2.5 mL deionized water, vortexed for 60 s (Vortex-Genie 2, Scientific Industries, Bohemia, NY, USA), and centrifuged for 20 min at 3220 g at room temperature. The supernatant was removed by decantation and drainage of the residual non-bound water by placing the centrifugation tube upside-down on filter paper for 60 min. WBC was calculated as:

$$\mathrm{WBC}=\frac{m_{1}-m_{0}}{m_{0}}$$

where m_0_ is the initial weight, m_1_ is the final weight. The oil binding capacity (OBC) was analysed using sunflower oil instead of deionized water. Except for the vortexing step (120 s), the experimental procedure was performed in analogy to the WBC assay. OBC was similarly calculated.

#### 2.5.2. Determination of Emulsion Capacity and Emulsion Stability

Emulsifying properties were determined according to the method of Mshayisa and van Wyk [26] The samples were dispersed in distilled water 1% (*w*/*v*), and 15 mL of the dispersion was homogenized with 15 mL of vegetable oil at a speed of 10,000 rpm for 3 min. Subsequently, the samples were centrifuged (Thermo Electron Corporation Jouan MR1812, Waltham, MA, USA) at 3220 g for 5 min and the volume of the individual layers were read. Emulsion stability was evaluated by heating the emulsion for 30 min at 80 °C. Then, the samples were centrifuged at 3200 g for 5 min. The emulsifying capacity (%) was expressed as a percentage of the volume of the emulsified layer (mL) against the volume of the whole layer (mL). Emulsion capacity and emulsion stability were calculated from the formula:

$$\%\;\mathrm{Emulsion}\;\mathrm{capacity}\;\left(\mathrm{EA}\right)=\frac{V_{e}}{V}\times 100$$

$$\%\;\mathrm{Emulsion}\;\mathrm{stability}\;\left(\mathrm{ES}\right)=\frac{V_{30}}{V_{e}}\times 100$$

where: V—total volume of tube contents, V_e_—the volume of the emulsified layer, V_30_—the volume of the emulsified layer after heating.

#### 2.5.3. Determination of Foam Capacity and Foam Stability

Foaming capacity (FC) and foam stability (FS) were determined according to the method of Zielinska et al. [27]. First, 20 mL of a 1% sample was homogenized in a high shear homogenizer mixer (Polytron PT 2500E, United Scientific, Cape Town, South Africa) at a speed of 10,000 rpm for 4 min. The whipped sample was then immediately transferred into a graduated cylinder. The total volume was read at time zero and 30 min after homogenization. The foaming capacity and foam stability were calculated from the formula:

$$\%\;\mathrm{Foaming}\;\mathrm{capacity}\;\left(\mathrm{FC}\right)=\frac{V_{0}-V}{V}\times 100$$

$$\%\;\mathrm{Foaming}\;\mathrm{stability}\;\left(\mathrm{FS}\right)=\frac{V_{30}}{V_{0}}\times 100$$

where: V—volume before whipping (mL), V_0_—volume after whipping (mL), V_30_—volume after standing (mL).

### 2.6. Determination of Antioxidant Activity

#### 2.6.1. Preparation of Edible Insect Extract

Two grams of the edible insect flours was mixed with 40 mL Milli-Q water in a 50 mL centrifuge tube. The edible insect flour solution was centrifuged (Thermo Electron Corporation Jouan MR1812, Waltham, MA, USA) at room temperature for 15 min at 8000 rpm, and the supernatant was collected and stored at 4 °C until further analysis and the pellet was discarded.

#### 2.6.2. Determination of DPPH Radical Scavenging Activity

The antioxidant activity of the extract was determined by the 1,1-diphenyl-2-picryl-hydrazyl radical scavenging (DPPH-RS) assay according to the method of Vhangani and van-Wyk [28]. The method uses a stable chromogen radical, DPPH in ethanol, which gives a deep purple colour. The reaction mixture was prepared by reacting 2 mL of edible insect extract with 4 mL of DPPH (0.12 mM) in 95% in ethanol. The reaction mixture was incubated for 30 min in the dark, and then the absorbance of the resulting solutions was measured at 517 nm using a spectrophotometer (Lambda 25, Perkin Elmer, Singapore). The control was prepared similarly, except that Milli-Q water was used, and TBHQ (0.1%) was used as a positive control. The percentage of inhibition was calculated using the formula:

$$\%\;\mathrm{DPPH}-\mathrm{RS}\;=\frac{A_{0\;\left(517\mathrm{nm}\right)-}A_{1\left(517\mathrm{nm}\right)}}{A_{0\left(517\mathrm{nm}\right)}}\times 100$$

where: A_0_ is the absorbance of the negative control (water) at 517 nm and A_1_ is the absorbance of the edible insect extract at 517 nm test sample.

#### 2.6.3. Determination of ABTS^+^ Radical Scavenging Activity

The experiment was performed according to the method of Chatsuwan et al. [29] and Mshayisa and van Wyk [26]. The 2,2-Azinobis (3-ethylbenzothiazoline-6-sulphonic acid) diammonium salt (ABTS^•+^) radical was produced by reacting 7.4 mM ABTS stock solution with 2.45 mM potassium persulphate at a ratio of 1:1 (*v*/*v*). The mixture was allowed to react for 12–16 h at room temperature in the dark. This working solution of ABTS^•+^ solution was diluted with 95% ethanol at a ratio of 1:50 (*v*/*v*) in order to obtain an absorbance of 1.00 at 734 nm. A fresh ABTS^•+^ solution was prepared daily for each assay. The reaction mixture contained 0.15 mL of edible insect extract solution and 2.85 mL of ABTS^•+^ solution. The mixture was incubated at room temperature for 6 min in the dark. Then, the absorbance was measured at 734 nm in a spectrophotometer (Lambda 25, Perkin Elmer, Singapore). The control was prepared in the same manner, except that distilled water was used instead of the sample, and TBHQ (0.1%) was used as a positive control. The scavenging activity was determined according to the equation:

$$\%\;\mathrm{ABTS}-\mathrm{RS}=\frac{A_{control\left(730mn\right)}-A_{sample\left(730nm\right)}}{A_{control\left(730nm\right)}}\times 100$$

where: $A_{control}$ is the absorbance of the control (water) at 730 nm and $A_{sample}$ is the absorbance of edible insect extract at 730 nm.

#### 2.6.4. Determination of Fe^2+^ Chelating Activity

The chelating effect on ferrous ions of the prepared extracts was estimated by the method of Sudan et al. [30] with slight modifications. Briefly, 1 mL of each edible insect extract was mixed with 1.85 mL of Milli-Q water and 0.05 mL of 2 mM FeCl_2_. Next, the reaction was initiated by the addition of 0.1 mL of 5 mM ferrozine into the mixture, which was then left at room temperature for 10 min and the absorbance of the mixture was determined at 562 nm using a spectrophotometer (Lambda 25, Perkin Elmer, Singapore). The percentage of chelating activity was calculated as follows:

$$\%\;\mathrm{Chelating}\;\mathrm{activity}\;\frac{A_{0}-A_{1}}{A_{0}}\times 100$$

where: A_0_ is the absorbance of the negative control (water) control and A_1_ is the absorbance of the edible insect extract.

#### 2.6.5. Determination of Reducing Power

The reducing power was determined according to the method of Athukorala et al. [31]. First, 1.0 mL aliquots of edible insect were mixed with 2.5 mL of phosphate buffer (0.2 mM, pH 6.6) and 2.5 mL of potassium ferricyanide. The reaction mixture was vortexed for 10 s and thereafter incubated at 50 °C in the water bath for 20 min. Thereafter, 2.5 mL of 10% trichloroacetic acid (TCA) was added to the reaction mixture, and then vortexed for 10 s, 2.5 mL of the solution was then pipetted out into beakers and mixed with 2.5 mL of distilled water and 0.5 mL of FeCl_3_ was added and absorbance was measured at 700 nm in a spectrophotometer (Lambda 25, Perkin Elmer, Singapore).

### 2.7. Statistical Analysis

All assays were performed in triplicates, and the obtained data were presented as means ± standard deviation. Statistical analysis was performed by testing significant differences (*p* < 0.05) between treatments using multivariate analysis of variance (MANOVA), and Duncan’s multiple range test was used to separate means where differences existed. Principal Component Analysis (PCA) was applied to extract the components that explained the variability in the edible insect flours antioxidant and functional properties. All quantitative data were analysed using SPSS 27.0 (2005) (SPSS Inc., Chicago, IL, USA).

## 3. Results and Discussion

### 3.1. Proximate Composition of Edible Insect Flours

The proximate composition of edible insect flours (*G. belina*, *H. illucens* and *M. subhylanus*) is depicted in Table 1. Protein is the dominant nutrient in all three edible insect flours, followed by crude fat. The protein content was significantly (*p* < 0.05) different between all the edible insect flours, and it ranged from 34.90–52.74%. This is superior to other protein sources, such as beef, eggs, milk, and soybeans, where protein constitutes approximately 30 and 45% of dry matter [32]. The protein content of *H. illucens* (34.90%) was significantly lower (*p* < 0.05) compared to *M. subhylanus* (52.74%). Our findings agreed with the results reported by Bußler et al. [11] on *H. illucens* (34.70%). In a literature review study conducted by Meyer-Rochow et al. [33] the protein content of the Macrotermes species ranged from 20.4–39.7%. Moreover, Kwiri et al. [34] reported the protein content of *G. belina* to be (55.41%). These values are higher than the result obtained in this study of the same insect flour. The differences in protein content can be attributed to differences in the edible insect flour, level of individual development, sex, feed type, climate, and geographical location. In this way, the edible insect flours are diversified nutritionally. The edible insects reported in this study may offer an affordable source of protein, especially for low-income communities and be used as ingredients in flour form to minimise the aversion towards consuming insects [35,36].

The ash content of *G. belina* (11.38%), *H. illucens* (7.46%) and *M. subhylanus* (6.38%) was higher than the values reported for *M. nigeriensis* (3.24%) by Omotoso [37]. However, the values were comparable to those of *Macrotermes bellicosus* (*M. bellicosus*) (11.83%) reported by Adepoju and Omotayo [38]. Torruco-Uco et al. [39] also reported the ash of *Sphenarium purpurascens* (*S. purpurascens*) to be 2.31–3%, the values are much lower than the values reported in this study. Nyakeri et al. [40] reported *H. illucens* to contain 14.61% ash which is higher than the value of the similar species in this study which had 7.46% ash content. Considerable levels of ash indicate that the samples are a good source of minerals. The variation among the ash contents of samples may be driven by the difference in location, diet, and season in which the insects are reared and harvested [41]. Therefore, the addition of edible insect flour in processed food products has the potential to enhance the mineral content of food, especially where food fortification is essential. The considerable good ash content of the edible insect flours signifies good mineral composition that the edible insect flours might contain [42].

As shown in Table 1, the moisture of the three edible insect flours ranged from 5.77–6.59% and no significant differences (*p* > 0.05) were observed amongst all the edible insect flours. Siulapwa et al. [43] reported the moisture content of *G. belina* to be 9.1%, which is higher than the value reported in this study. Moreover, Anaduaka et al. [36] also reported high moisture values for *Zonocerus variegatus (Z. variegatus)* and *Oryctes rhinoceros* larva (*O. rhinoceros* larva) to be 11.85–26.17%, respectively. The low moisture values obtained in this study suggest that it likely results in low water activity and, therefore, can potentially extend the shelf-life of insect flours.

As illustrated in Table 1 the crude fat content in *G. belina*, *H. illucens*, and *M. subhylanus* was 13.91, 27.92 and 6.35%, respectively. *H. illucens* (27.92%) results were higher than those reported by Payne et al. [44] of the similar species (14%). Ganguly et al. [45] reported the fat of Oxya chinensis to be 2.2%, which is lower than the results obtained in this study. Moreover, Melo et al. [46] reported *S. purpurascens* to be 5.75%, which is comparable to *M. subhylanus.* However, Sogbesan and Ugwumba [47] reported the fat of *M. subhylanus* to be 10.6–22.2%, which is higher than the values of the similar species in this study. Fat is a major source of fuel in the body, and it is essential in the cell structures as well as in supplying some oil-soluble vitamins, such as vitamins A, D, E, K.

As the primary source of fibre and calories for humans, carbohydrates are essential components of proper nutrition [48]. The three edible insect flours (*G. belina*, *H. illucens* and *M. subhylanus)* showed no significant difference (*p* > 0.05) in their carbohydrate content and ranged from 22.33–28.10%, respectively. The observed carbohydrate content is low in comparison with those reported by Mishyna et al. [49] for *Schistocerca gregaria* (*S. gregaria*) and *Apis mellifera* (*A. mellifera*) flours which contained 47.2 and 54.10% carbohydrates, respectively.

Energy is primarily derived from carbohydrates, proteins, and fats in food, and because edible insects are high in these macromolecules, they have a high energy content [45]. As shown in Table 1 the energy values obtained for the edible insect flours ranged from 379.91–485.58 kJ. No significant differences (*p* > 0.05) were observed for *G. belina* and *M. subhylanus*. However, *H. illucens* was significantly different (*p* < 0.05) from the other two edible insect flours. The results reported in this study are similar to those reported by Montowska et al. [35] on edible insect flours of 486–524 kcal/100 g. Siulapwa et al. [43] reported *G. belina* energy values of (385 kcal/100 g), which is in the same range as *G. belina* energy value reported in this study.

### 3.2. Physicochemical Properties

#### 3.2.1. Colour Properties of Edible Insect Flours

**Figure 1:**
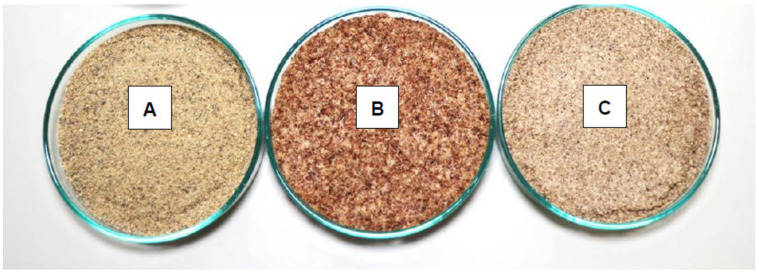
Ground edible insect flour of three different species. (**A**) *G. belina*; (**B**) *M. subhylanus*; and (**C**) *H. illucens*.

The colour attributes of edible insect flours measured were lightness (L*), greenness (−a*), redness (+a*), blueness (−b*), and yellowness (+b*). Lightness is the luminous intensity of colour measured on a scale of 0 to 100, with 0 indicating black and 100 indicating white [50]. Colour is a crucial factor influencing the acceptance of edible insects [18]. The descriptive colour determination of the three edible insect flours *G. belina*, *H. illucens* and *M. subhylanus* is shown in Table 2. There was a significant difference (*p* < 0.05) in the lightness of the edible insect flours, with *G. belina* (57.95) being the lighter in colour. No significant difference (*p* > 0.05) was observed in the redness of the three edible insect flours; however, *M. subhylanus* (5.72) was redder compared to *G. belina* (3.92) and *H. illucens* (4.46), respectively, as depicted in Figure 1.

#### 3.2.2. Bulk Density

Among other vital properties of powder products, bulk density (BD) has significant economic and functional importance, for example, in reducing packaging costs [51]. It is determined by particle density, internal porosity, and particle arrangement in the container [52]. Table 2 represents the bulk density of the three edible insect flours (*G. belina*, *H. illucens* and *M. subhylanus*). The bulk density of the edible insect flours varied from 0.51–0.64 g/mL and no significant difference (*p* > 0.05) was observed. Akpossan et al. [53] reported higher BD for *Imbrasia oyemensis* (*I. oyemensis*) to (1.1 g/mL), while in a study by [54] on *Imbrasia belina* (*I. belina*) the BD (0.65 g/mL) was comparable to that found in the present study. An apparent correlation exists between the bulk density and the protein content. Thus, the edible insect flours all had a low BD due to high protein content. Low BD of the flours is advantageous when storability and transportation are considered since the products could be easily transported and distributed [55]. Low BD flours also find application in the preparation of complementary foods and among the traditional techniques.

#### 3.2.3. Water Activity and pH of Edible Insects

**Figure 2:**
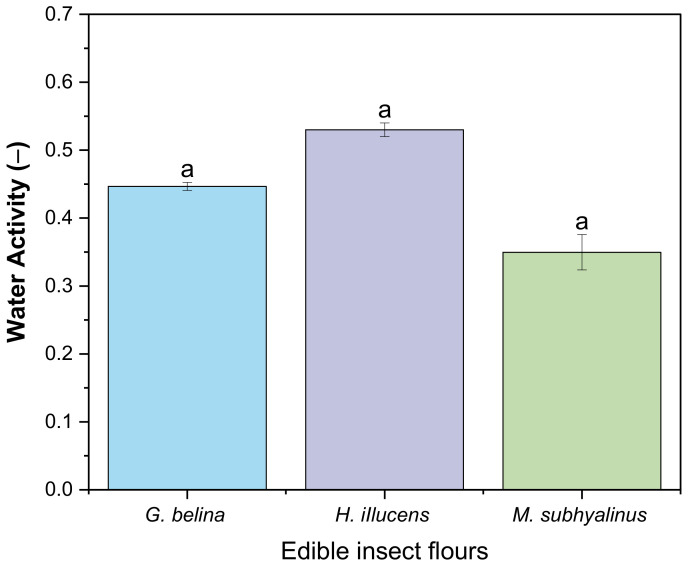
Water activity of three edible insect flours. Values are mean ± standard deviation, means with different superscripts are significantly different (*p* < 0.05).

Water activity is a measure of how efficiently the water present can take part in a chemical (physical) reaction or the water available enough for microbial growth to occur in a food product [56,57]. Generally, food deterioration due to microbial growth (yeast and moulds to pathogens) occurs at a range of 0.6 to 1.0 [57]. The water activity of the three edible insect flours *G. belina*, *H. illucens*, and *M. subhylanus* is depicted in Figure 2. The A_w_ of the edible insect flours ranged from *M. subhylanus* (0.35 ± 0.26), *G. belina* (0.45 ± 0.01), to *H. illucens* (0.53 ± 0.01), and there were no significant differences (*p* > 0.05) within the different edible insect flours. This implies that the edible insect flours are not susceptible to microbial growth. However, some enzymatic reactions, such as browning, transpire at the range of 0.3 to 1.0 and increase rapidly at 0.6 to 0.8. In this study, *M. subhylanus* had the lowest A_w_; therefore, it might be susceptible to enzymatic reactions rapidly compared to the other two edible insect flours.

In addition, pH in food contributes to reducing the growth of microorganisms, thereby ensuring food safety. The pH of *H. illucens* (8.93) had a significant difference (*p* < 0.05) between the pH of *G. belina* (6.12) and *M. subhylanus* (6.14), while there was no statistical difference (*p* > 0.05) between the pH of *G. belina* and *M. subhylanus* (Table 2). Lucas-González et al. [18] reported similar results for *Acheta domesticus* flour (6.31–6.48). The pH of these edible insect flours provides essential information since it determines which type of food matrix they can be added into without affecting their technological behaviour. Thus, potential food ingredients with pH values close to neutrality, such as those obtained in this study, will be better suited for application to neutral food matrices, such as meat replacers and baked products.

### 3.3. Techno-Functional Properties

#### 3.3.1. Water Binding Capacity and Oil Binding Capacity

Water binding capacity (WBC) and oil binding capacity (OBC) are critical features of food ingredients in food processing and applications. They are related to the ability to take up and retain water and oil, respectively, which directly affect the texture and the flavour of the products, especially in meat and bakery [58]. There are several intrinsic factors affecting the water-binding properties of food flours with relatively high protein. These include amino acid composition, protein conformation, and surface polarity/hydrophobicity [53]. Table 3 depicts the water binding capacity of the edible insect flours. Higher WBC was notable for *M. subhylanus* (1.46 g/g); however, there was no significant difference (*p* > 0.05) between this edible insect flour and that of *G. belina* (1.30 g/g). While the lower WBC value was observed for *H. illucens* flour (1.11 g/g), Zielinska et al. [27] reported higher WBC of *Schistocerca gregaria* (*S. gregaria*) (2.18 g/g). Similarly, Lucas-González et al. [18] reported the WBC of *Acheta domesticus* flour to be (3.82 g/g). However, the WBC of *M. subhylanus* (1.46 g/g) was higher than that reported for *T. molitor* (0.4 g/g). The apparent difference in the WBC could be due to the higher protein content in the *M. subhylanus*, which contains more hydrophilic groups to bind to water molecules. The WBC of the edible insect flours is comparable to plant-based flours, such as wheat and rice, which were reported to have WBC from 1.4–1.9 g/g [59]. This information is crucial for the application of these flours in the food industry. The significant difference in water holding capacity between the edible insect flours might be an indication of the different applications they might have in food. This is the first study to report on the WBC of edible insects, such as *G. belina* and *M. subhylanus*, to our knowledge.

The OBC is shown in Table 3. No significant difference was found (*p* > 0.05) between *H. illucens* (1.35 g/g) and *M. subhylanus* (1.48 g/g), and the lowest value was obtained for *G. belina* (0.89 g/g). These values are lower than those reported for *Gryllidae* sp. (2.02 g/g), *G. sigillatus* (2.82 g/g) and *A. domesticus* (3.37–3.52 g/g) [39]. Assielou et al. [60] reported the OBC of *O. owariensis* larvae flour to be 265.90% (2.65 g/g), which is higher than the OBC in this study. The OBC refers to the ability of the proteins in flour to physically bind to fat through capillary action, which is of great importance because fat is a flavour retainer and increases our ability to taste food. Akubor and Eze [61] illustrated that OBC has proven useful in the formulation of bakery products and sausages, and this shows that the studied flours (*M. subhylanus*, *H. illucens*, and *G. belina*), since they are low in OBC are, therefore, low flavour retainers and therefore may be useful in food systems that do not require high WBC/OBC values.

#### 3.3.2. Emulsion Capacity and Emulsion Stability

Proteins are surface-active agents that can form and stabilise the emulsion by creating electrostatic repulsion on the oil droplet surface. Generally, the emulsifying activity of proteins is affected by their molecular weight, hydrophobicity, conformation stability, surface charge, and physicochemical properties, such as pH, ionic strength, and temperature [62]. The results obtained for emulsion capacity (EC) and emulsion stability (ES) of the edible insect flours are presented in Table 3. The emulsion capacity of *G. belina*, *M. subhylanus*, and *H. illucens* were 41.76, 45.44, and 67.33%, respectively. The results for EC in this study are higher than those reported by Mishyna et al. [49] for *S. gregaria* (39.5%) and *A. mellifera* (20.8%) insect flours. The protein emulsification properties are known to be influenced by their surface hydrophobicity, which affects the protein’s ability to adsorb to the oil side of the interface. Higher emulsion capacities are usually associated with greater disintegration [11]. *M. subhylanus* had the highest EC (61.69%), which agrees with the macronutrient composition reported in Table 1.

In this study, the ES of *G. belina* (33.75%) and *M. subhylanus* (32.80%) were not significantly different (*p* > 0.05). The results are lower than those reported by Akpossan et al. [53] on *I. oyemensis* (84.76%). ES of *H. illucens* (42.45%) was comparable to that of the larva of *Cirina* (45.36%) reported by Omotoso. Adebowale et al. [63] reported adequate emulsification but poor stability in African cricket (*Gryllidae* sp.) flour. Food manufacturers have a growing demand for sustainable and secure protein sources. Currently, the most widely used emulsifiers are casein and whey [16]. Therefore, edible insect flours’ high emulsion capacity and stability highlight the potential to effectively utilise them in food emulsions.

#### 3.3.3. Foam Capacity and Foam Stability

Foams are colloidal systems that consist of a continuous aqueous phase and a dispersed gas phase [16]. Foam formation is governed by the transportation, penetration, and reorganisation of molecules at the air-water interface. To exhibit good foaming properties, a protein must be capable of migrating rapidly to the air-water interface, unfolding, and rearranging at the interface. Table 3 displays the FC and FS of the edible insect flours. The FC was higher for *G. belina* (5.81%); however, no significant differences (*p* > 0.05) were observed amongst all three edible insect flours. The FC values reported by Torruco-Uco et al. [39] for *Gryllidae* sp. (6%) were comparable to the reported values in this study. Zielinska et al. [27] reported FC of *G. sigillatus* (41%) while Assielou et al. [60] reported *Oryctes owariensis* (*O. owariensis*) larvae to have FC of (17.87%), which is also higher than the values reported in this study. This study shows that the low FC can be related to highly ordered globular proteins that resist surface denaturation [53,64].

There were no significant differences (*p* > 0.05) in FS of the edible insect flours in this study. However, the results obtained were higher than those reported by [54] on *I. belina* larvae flour (1.4–5.1%), whereas Omotoso [65] reported *Cirina forda* larva FS to be 3.00%, which is much lower than the FS reported in this study. There was a notable significant difference between the FC and FS values of the edible insect flours, and these results indicate that the proteins and other components of the edible insect flours have a greater ability to form a strong and cohesive film around air bubbles and greater resistance of air diffusion from the bubbles [66].

Presently, research is focused on finding alternatives to eggs, which are commonly used as a foaming agent in food products [16]. The data presented in this study showed that the three edible insect flours (*G. belina. H. illucens* and *M. subhylanus*) exhibited excellent foaming properties; hence, they can be a suitable foaming agent and has potential for such food applications.

### 3.4. Antioxidant Properties

#### 3.4.1. DPPH-RS of Edible Insect Flours

**Figure 3:**
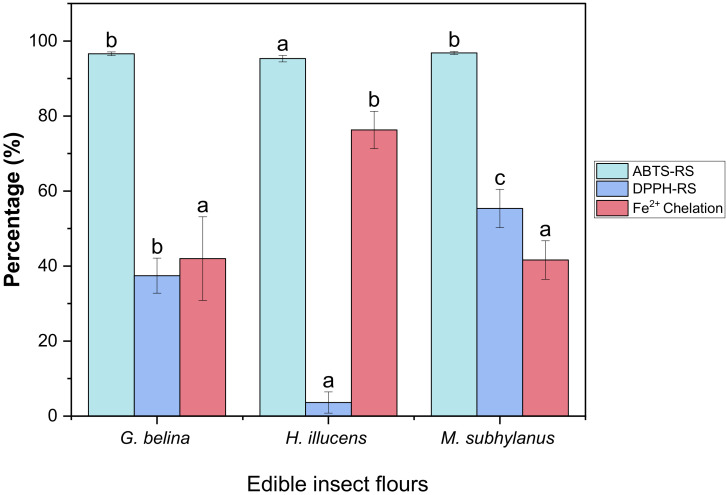
Scavenging effect of DPPH-RS, ABTS-RS and Fe^2+^ chelating activity of edible insect flours. Values are mean ± standard deviation; means with different superscripts are significantly different (*p* < 0.05).

The DPPH radical-scavenging (DPPH-RS) assay is a widely used method for evaluating the ability of food matrices to scavenge free radicals generated from the DPPH reagent, which undergo SET mechanism [67]. DPPH is a stable free radical that shows maximum absorbance at 517 nm in ethanol and changes from purple to yellow in the presence of antioxidants. When a DPPH radical encounters an electron-donating substrate, such as an antioxidant, the radical is scavenged [68]. As illustrated in Figure 3, the insect flours differed significantly (*p* < 0.05) from one another, with *M. subhylanus* (55.57%) exhibiting the highest radical scavenging activity followed by *G. belina* (37.44%) and *H. illucens* (3.63%), respectively. In a study reported by Navarro del Hierro et al. [69] *T. molitor* and *A. domesticus* extracts, the DPPH-RS was 57 and 72%, respectively, and the values for *T. molitor* are comparable to those of *M. subhylanus* from this study. Nabil et al. [70] also reported on *Moroccan cladode* flour, and the radical scavenging activity was between 7.18 and 72.37%, which is in line with the radical scavenging activity reported in this study. The results, therefore, suggest that the edible insect flours could be scavenging agents and imply that they have the ability to react with free radicals. This study supports the observation of Mshayisa and van-Wyk [26], who proposed that edible insects can be used as novel functional components in food compositions.

#### 3.4.2. ABTS-RS of Edible Insect Flours

The ABTS^+^ radical scavenging activity was determined to assess the antioxidant potential of *H. illucens*, *G. belina* and *M. subhylanus*. As depicted in Figure 3, no significant differences (*p* > 0.05) were observed between *M. subhylanus* (96.81%) and *G. belina* (96.61%). However, a significant difference (*p* < 0.05) was observed between the two edible insect flours compared to *H. illucens* (95.32%). It was also observed that *H. illucens* showed lower DPPH-RS as compared to ABTS-RS. The difference in scavenging patterns of ABTS-RS and DPPH-RS could be responsible for these observations. ABTS is more accessible to hydrophilic peptides, while hydrophobic peptides can interact easily with peroxyl radicals, such as DPPH [71]. Most importantly, to our knowledge, this is the first study to establish the antioxidant indices of these three edible insect flours. This study’s findings have implications for the utilization of edible insect flours as functional components in food.

#### 3.4.3. Metal Chelation of Edible Insect Flours

The chelation of Fe^2+^ was used to determine the ability of edible insect flours in metal-chelating activity. Ferrozine quantitatively forms complexes with Fe^2+^ ions in the presence of chelating agents, the development of complexes is slowed in the presence of chelating substances disrupted, resulting in the decrease in colour formation [68]. As shown in Figure 3, all edible insects had a high ability to chelate Fe^2+^. In this study, the highest chelating ability activity was observed in *H. illucens* (76.30%). Moreover, there were no significant differences (*p* > 0.05) in *G. belina* (42.00%) and *M. subhylanus* (41.61%). Ferrous ion (Fe^2+^) is the most potent pro-oxidant among metal ions. This ion can interact with hydrogen peroxide in a Fenton reaction to produce the reactive oxygen species and hydroxyl free radical (OH), leading to the initiation and/or acceleration of lipid oxidation in food [72]. Therefore, the ability of these edible insect flours to chelate Fe^2+^ suggests they can reduce or avoid the free radical formation. To the best of our knowledge, this is the first study to empirically investigate the Fe^2+^ chelation of edible insect flours, such as *G. belina* and *M. subhylanus.* The results of this study are vital since they indicate that edible insect flours possess considerable meatal chelating activity, which is critical in antioxidant activity since it reduces the concentration of transition metals that catalyse lipid oxidation.

#### 3.4.4. Reducing Power of Edible Insect Flours

**Figure 4:**
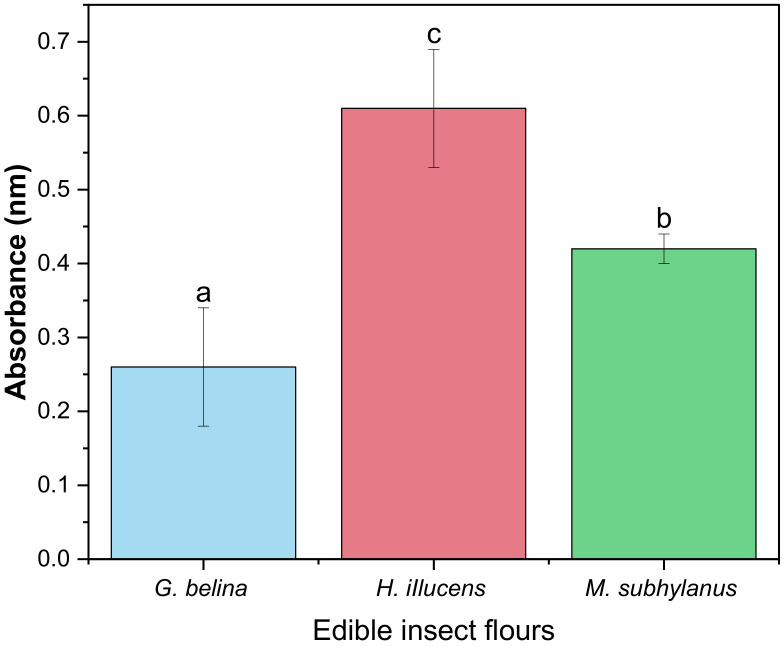
Reducing power activity of edible insect flours. Values are mean ± standard deviation; means with different superscripts are significantly different (*p* < 0.05).

Reducing power is a useful indicator of food component antioxidant activity. In this test, the ferric chloride/ferric cyanide complex is reduced to ferrous form (Fe^2+^) in the presence of antioxidants, allowing the Fe^2+^ concentration to be measured spectrophotometrically by measuring the Prussian blue colour produced at 700 nm [73]. The reducing power assay is often used to evaluate the ability of antioxidants to donate an electron to the free radical [74]. In this study, the ability of edible insect flours to reduce Fe^3+^ to Fe^2+^ was investigated, and the results are depicted in Figure 4. A significant difference (*p* < 0.05) was observed between all the edible insect flours. *H. illucens* (0.61) had the highest RP, while *G. belina* (0.26) had the lowest RP. As articulated by Zielińska and Pankiewicz [75], due to their high protein nature, edible insects are, therefore, potential sources of bioactive proteins that could also possess antioxidant activity. In addition, due to the high reducing power, the obtained results suggest that *H. illucens* soluble proteins contain amino acids or peptides that act as electron donors and can react with free radicals to transform them into stable compounds.

### 3.5. Principal Component Analysis

**Figure 5:**
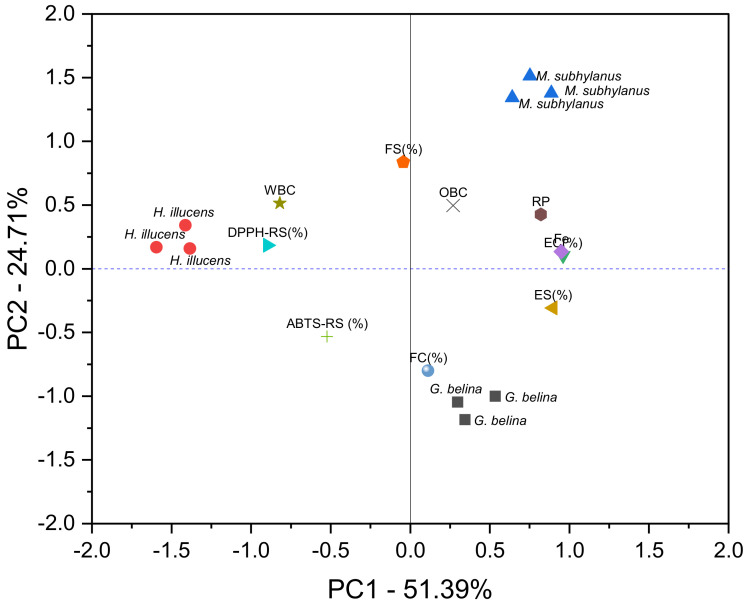
Principal components analysis plot for techno-functional properties and antioxidant indices of edible insect flours.

Principal component analysis (PCA) was performed to understand the inter-relationships among the measured techno-functional properties and antioxidant activity indices and the similarities and differences among the edible insect samples. The suitability of data reduction by PCA was established by several factors, such as the high correlations between the variables (correlation matrix) and the significant (*p* ≤ 0.05) Bartlett’s test, as well as the Kaiser–Meyer–Olkin measure (0.68), which was significantly higher than the recommended minimum of 0.6. The PCA results were displayed using score and loading plots (Figure 5). To determine the relative contributions of the principal components in overall total variability, only the eigenvalues greater than one were considered. Thus, the first three principal components (PC1, PC2 and PC3) were found to be significant and explained 87.99% variability in the data set (Table S1). Component 1 accounted for 51.39% of the total variability, and represented EC (0.960), Fe Chelation (0.949), ES (0.897) and DPPH-RS (−0.897) while FS (0.837), FC (−0.080), ABTS-RS (−0.531) and WBC (0.515) contributed to PC2, with a total variability contribution of 24.71%. The PC3 accounted for 11.89% of the total variability due to OBC (0.745), FC (0.504), ABTS-RS (0.442) and RP (0.247), respectively, as shown in Table S1 (Supplementary Materials). The edible insects were clearly distributed into three clusters (Figure 5). It can be seen that *M. subhylanus* can be separated from *H. illucens* based on the DPPH-RS, WBC, and foam stability. In Figure 5, *H. illucens* were grouped in close proximity with values of component 1, whereas *M. subhylanus* and *G. belina* are diametrically opposed in PC2 (meaning they are on the negative and opposite sides). PCA showed that *M. subhylanus* and *G. belina* located on the opposite sides of PC2, the FS, OBC and FC were to be majorly responsible for the difference in the edible insect flours. This was due to the high FC and OBC exhibited by *M. subhylanus* samples, while *G. belina* exhibited the lowest OBC. Therefore, PCA could be helpful to provide valuable information on the classification and discrimination of edible insect flours and on relationships between antioxidant indices and techno-functional properties.

## 4. Conclusions

This study was undertaken to establish the potential for edible insect flours as a source of nutrients, as well as their techno-functional and antioxidants properties. The studied edible insect flour species were rich in protein and fat, which are essential nutrients required for the human diet. The results obtained for the physicochemical properties make the flours valuable to the food industry as potential fortifiers, such as *G. belina*, which was yellower and redder in colour since this characteristic is of importance in instances where a noticeable colour change to the product is not desired. *M. subhylanus* exhibited good water binding capacity, and the flour was generally found to have superior techno-functional properties among the studied species. This makes it useful for producing foods such as sausages and bakery products. The studied edible insects have unique techno-functional properties that can be exploited to provide functional ingredients. Future studies on the shelf life, rheological and structural properties of the edible insect flours are essential prior to incorporation in food product formulations.

## Figures and Tables

**Table 1:** Proximate composition of three edible insect flours.

Edible Insects	Crude Protein (%)	Ash (%)	Moisture (%)	Crude Fat (%)	Carbohydrates (%)	Energy (%)
*G. belina*	46.70 ± 0.82 ^b^	11.38 ± 2.20 ^b^	5.68 ± 0.25 ^a^	14.04 ± 0.12 ^b^	22.10 ± 1.45 ^a^	399.38 ± 6.03 ^a^
*H. illucens*	34.90 ± 0.47 ^a^	7.50 ± 1.65 ^a^	5.76 ± 0.01 ^ab^	27.93 ± 6.13 ^c^	23.66 ± 7.84 ^a^	485.58 ± 26.69 ^b^
*M. subhylanus*	52.74 ± 1.47 ^c^	6.41 ± 0.07 ^a^	6.40 ± 0.06 ^b^	6.36 ± 0.05 ^a^	27.27 ± 1.19 ^a^	379.91 ± 1.06 ^a^

Values are mean ± standard deviation. Means within a column followed by the same superscript are not significantly (*p* > 0.05) different.

**Table 2:** Physicochemical properties of three edible insect flours.

Edible Insects	L*	a*	b*	Bulk Density (g/mL)	pH
*G. belina*	57.95 ± 0.31 ^c^	3.92 ± 1.49 ^a^	20.02 ± 1.97 ^b^	0.65 ± 0.01 ^b^	6.12 ± 0.03 ^a^
*H. illucens*	53.69 ± 0.54 ^b^	4.46 ± 0.36 ^a^	13.08 ± 2.68 ^a^	0.51 ± 0.01 ^a^	8.93 ± 0.05 ^b^
*M. subhylanus*	43.52 ± 0.56 ^a^	5.72 ± 3.90 ^a^	12.00 ± 2.70 ^a^	0.64 ± 0.00 ^b^	6.14 ± 0.02 ^a^

Values are mean ± standard deviation. Means within a column followed by the same superscript are not significantly (*p* > 0.05) different.

**Table 3:** Techno-functional properties of three edible insect flours.

Edible Insects	WBC (g/g)	OBC (g/g)	EC (%)	ES (%)	FC (%)	FS (%)
*G. belina*	1.30 ± 0.12 ^ab^	0.89 ± 0.12 ^a^	41.76 ± 2.84 ^a^	33.75 ± 2.29 ^a^	5.81 ± 3.69 ^a^	95.32 ± 2.37 ^a^
*H. illucens*	0.11 ± 0.02 ^a^	1.35 ± 0.09 ^b^	67.33 ± 8.49 ^b^	42.45 ± 5.07 ^b^	5.69 ± 1.41 ^a^	97.38 ± 1.70 ^a^
*M. subhylanus*	1.46 ± 0.06 ^b^	1.48 ± 0.07 ^b^	45.44± 4.28 ^a^	32.80 ± 0.47 ^a^	4.71 ± 2.46 ^a^	97.51 ± 1.22 ^a^

Values are mean ± standard deviation. Means within a column followed by the same superscript are not significantly (*p* > 0.05) different. WBC: water-binding capacity, OBC: oil biding capacity, EC: emulsion capacity, ES: emulsion stability, FS: foam stability, and FC: foam capacity.

## Data Availability

The datasets used and/or analysed during the current study are available from the corresponding author on reasonable request.

## Supplementary Materials

The following supporting information can be downloaded at: https://www.mdpi.com/article/10.3390/foods11070976/s1. Table S1: Principal components for illustrating the interpretation in Figure 5.
