# Supplementary material for: Proximate, Physicochemical, Techno-Functional and Antioxidant Properties of Three Edible Insect (Gonimbrasia belina, Hermetia illucens and Macrotermes subhylanus) Flours
Source: Foods. 2022 Mar 28;11(7):976. doi: 10.3390/foods11070976 (PMC8997929; doi:10.3390/foods11070976)
Supplement: Supplementary file 1 [file foods-11-00976-s001.zip › foods-1609687-supplementary.pdf]

Table S1: Principal components for illustrating the interpretation in Figure 5**.**

Variables	PC1	PC2	PC3
EC(%)	.960	.104	.089
Fe Chelation (%)	.949	.134	-.181
ES(%)	.897	-.307	.125
DPPH-RS (%)	-.897	.184	.179
RP (abs nm)	.821	.426	.247
WBC ($g.g^{-1}$)	-.820	.515	.106
FS (%)	-.043	.837	.155
FC (%)	.112	-.800	.504
ABTS-RS (%)	-.524	-.531	.442
OBC ($g.g^{-1}$)	.269	.496	.745

Where, PC1, PC2 and PC3 are first three Principal components.
